# Analysis of adverse drug events in pulmonary *Mycobacterium avium* complex disease using spontaneous reporting system

**DOI:** 10.1186/s12879-022-07568-z

**Published:** 2022-06-29

**Authors:** Takuya Ozawa, Ho Namkoong, Risako Takaya, Yusuke Takahashi, Koichi Fukunaga, Yuki Enoki, Kazuaki Taguchi, Junko Kizu, Kazuaki Matsumoto, Naoki Hasegawa

**Affiliations:** 1 Division of Pulmonary Medicine, Department of Medicine, Keio University School of Medicine, Tokyo, Japan; 2 Department of Infectious Diseases, Keio University School of Medicine, 35 Shinanomachi, Shinjuku, Tokyo 160-8582 Japan; 3 Division of Practical Pharmacy, Faculty of Pharmacy, Keio University, Tokyo, Japan; 4 Division of Pharmacodynamics, Faculty of Pharmacy, Keio University, Tokyo, Japan

**Keywords:** *Mycobacterium avium* complex (MAC), Adverse events, Clarithromycin, Ethambutol, Rifampicin, Reporting odds ratio, Weibull distribution, Japanese Adverse Drug Event Report (JADER)

## Abstract

**Background:**

In Japan, *Mycobacterium avium* complex lung disease (MAC-LD) is the most common in nontuberculous mycobacterial lung disease. Patients often experience adverse events, resulting in the discontinuation of treatment, which causes treatment failure. The JADER (Japanese Adverse Drug Event Report) database is a database of adverse events that allows us to collect real-world data on adverse events. We can collect large-scale data cost-effectively and detect signals of potential adverse events such as reporting odds ratio (ROR) by using spontaneous reporting systems. In this study, we aimed to elucidate the adverse events of clarithromycin (CAM), ethambutol (EB), and rifampicin (RFP) using the JADER database.

**Methods:**

We included cases of MAC-LD between April 2004 and June 2017. We investigated sex, age, and medications that may have caused the adverse events, outcomes, and time of onset. We calculated the safety signal index as the ROR. Time-to-event analysis was performed using the Weibull distribution.

**Results:**

The total number of adverse events of CAM, EB, and RFP was 2780, with 806 patients. In the overall adverse events, hematologic and lymphatic disorders were the most common adverse events, with 17.3%, followed by eye disorders (16.6%), and hepatobiliary disorders (14.0%). The outcomes were as follows: recovery, 40.0%; remission, 27.1%; non-recovery, 11.2%; and death, 7.1%. Regarding the most common onset time of CAM, EB, and RFP was within 120 days at 40%, 181–300 days at 43.6%, and within 120 days at 88.5%. For CAM, the RORs of infections and infestations, hepatobiliary system disorders, and immune system disorders were 4.13 (95% confidence interval [CI], 2.3–7.44), 2.61 (95% CI, 1.39–4.91), and 2.38 (95% CI, 1.04–5.44). For EB, the ROR of eye disorders was 215.79 (95% CI, 132.62–351.12). For RFP, the RORs of renal and urinary tract disorders and investigations were 7.03 (95% CI, 3.35–14.77) and 6.99 (95% CI, 3.22–15.18). The β value of EB was 2.07 (95% CI, 1.48–2.76), which was classified as a wear-out failure type.

**Conclusions:**

For MAC-LD, the adverse event which has the highest ROR is infections and infestations in CAM, eye disorders in EB, renal and urinary tract disorders in RFP. Adverse events of EB occur after 180 days, whereas the adverse events of CAM and RFP occur early in the course of treatment.

**Supplementary Information:**

The online version contains supplementary material available at 10.1186/s12879-022-07568-z.

## Background

The incidence of nontuberculous mycobacterial lung disease (NTM-LD) is increasing worldwide [1]. In Japan, *Mycobacterium avium* complex lung disease (MAC-LD) is the most common NTM-LD [2, 3], and its mortality rate is increasing [4]. MAC-LD generally progresses slowly in immunocompetent hosts, causes muscle weakness, and affects their quality of life [5, 6].

In Japan, clarithromycin (CAM), ethambutol (EB), and rifampicin (RFP) are the standard regimen for MAC-LD. This is the same regimen for MAC bacteremia in the late stages of HIV [7, 8]. Patients often experience adverse events during long-term pharmacological therapy for MAC-LD, resulting in the discontinuation of treatment [9–13], which causes treatment failure [14]. Therefore, we need to understand the actual situation of adverse events to improve the therapeutic outcome of MAC-LD. However, it is difficult to examine trends in real-world conditions because of the length of time to follow patients efficiently.

In Japan, a system for reporting adverse events suspected to be caused by pharmaceutical products has been established. The Pharmaceuticals and Medical Devices Agency (PMDA) has published an adverse event database, JADER (Japanese Adverse Drug Event Report), which can be viewed on its website. It has been noted that we can collect large-scale data cost-effectively and detect signals of potential adverse events such as reporting odds ratio (ROR) by using spontaneous reporting systems [15]. ROR is the proportion of spontaneous reports about a drug that is related to a specific adverse event divided by the proportion of corresponding adverse events to all other drugs. The JADER allows us to collect real-world data on adverse events. Several studies using the JADER database have been reported on various diseases.

Few studies have summarized the adverse event reports of MAC-LD treatment in Japan in general. In this study, we summarized the adverse events of clarithromycin (CAM), ethambutol (EB), and rifampicin (RFP) using the JADER database; investigated sex, age, outcome, and onset; and calculated ROR and Weibull distribution. We aimed to understand the actual adverse events associated with MAC-LD.

## Methods

### Study population

JADER data were downloaded from the PMDA website (http://www.info.pmda.go.jp/fukusayoudb/CsvDownload.jsp) in November 2018. We included cases of MAC-LD as primary illness between April 2004 and June 2017. Duplicate data were identified by the same identification number and outcome and were excluded. Patients with NTM-LD were excluded from the study because those patients may have diseases other than MAC-LD. The JADER dataset consists of four tables containing the following information: (1) patient information, including sex, age, and body weight; (2) patient drug information; (3) patient adverse events and outcomes; (4) medical history and primary illness. We extracted the study data from these tables for our analyses. We investigated sex, age, and medications that may have caused the adverse events, outcomes, and time of onset. Patients without data on age, sex, and outcomes were classified as unknown. We used the date of onset of the adverse events in our analyses. Cases in which only the year was described were excluded. In cases where the year and month were described but the day was not, the day was set to 15. Reports with unknown dates were excluded from the Weibull analysis. We excluded cases with concomitant medications from the analysis of ROR.

This study was conducted in accordance with the Guidelines for the Conduct of Pharmacoepidemiological Studies in Drug Safety Assessment with Medical Information Databases [16].

### Statistical analysis

We calculated the disproportionate reporting as the ROR using the standard method with a 2 × 2 contingency table. We performed the disproportionality analysis using the System Organ Class MedDRA level, and grouped MedDRA preferred terms into System Organ Class because of low numbers of cases. If the lower limit of the 95% confidence interval (CI) was greater than 1.0, the drug was considered single-positive. We assumed that the time-to-event data followed a Weibull distribution with shape parameter β, representing the failure rate estimate. If β < 1, the reporting of adverse events decreases with time and is classified as an early failure type. If β = 1, reporting of adverse events appears at a constant rate and is classified as an accidental failure type. If β > 1, reporting of adverse events increases with time and is classified as a wear-out failure type. The number of days until adverse events was calculated by dividing the number of days by 30 days. If the onset of adverse events was more than 1095 days (3 years) after the start of treatment, the onset time was 1095 days. We used JMP 11 (SAS Institute Inc., Cary, NC, USA) for the analysis.

## Results

**Fig. 1:**
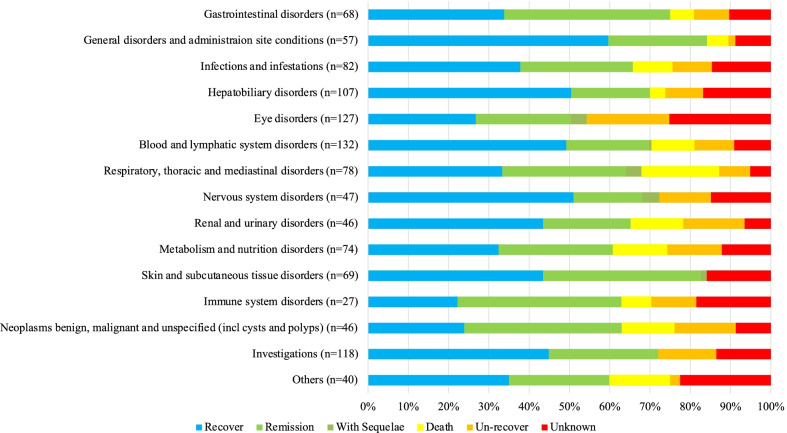
Outcome of adverse events with at least 20 cases in all three drugs: clarithromycin, ethambutol, and rifampicin. Vertical axis shows the types of adverse events. Horizontal axis shows the proportion of cases by adverse event. Each bar chart is divided by the number of cases according to outcome

The total number of adverse events of CAM, EB, and RFP for MAC-LD registered in the JADER database in November 2018 was 2780, with 806 patients. Of the 2780 cases, 1200 outcomes were found in 763 cases. Figure 1 shows the percentage of overall adverse events with at least 20 cases and outcomes. Blood and lymphatic system disorders were the most common adverse events, with 17.3% (132/763), followed by eye disorders (16.6%, 127/763), and hepatobiliary disorders (14.0%, 107/763). The outcomes were as follows: recovery, 40.0% (481/1200); remission, 27.1% (325/1200); non-recovery, 11.2% (134/1200); and death, 7.1% (85/1200). For each adverse event, death occurred most in respiratory, thoracic, and mediastinal disorders at 19.2% (15/78), followed by metabolism and nutrition disorders at 13.5% (10/74).

### Clarithromycin

**Fig. 2:**
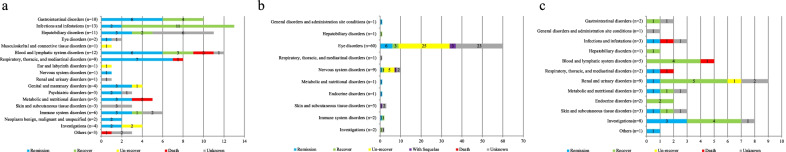
Outcome of adverse events in each drug. Vertical axis shows the types of adverse events. Horizontal axis shows the number of patients who experienced adverse events by the type of them. Each bar chart is divided by the number of patients according to outcome. **a** Clarithromycin. **b** Ethambutol. **c** Rifampicin

**Fig. 3:**
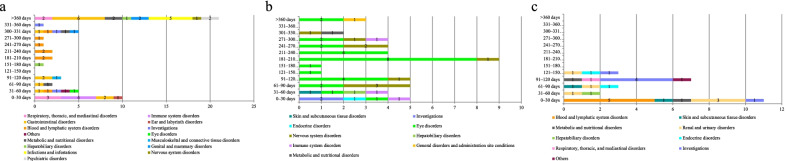
Onset of adverse events in each drug. Vertical axis shows the onsets of adverse events. Horizontal axis shows the number of patients who experienced adverse events by onset. Each bar chart is divided by the number of patients according to the type of adverse event. **a** Clarithromycin. **b** Ethambutol. **c** Rifampicin

Infections and infestations were the most common adverse events at 16.9% (13/77), followed by blood and lymphatic system disorders at 16.9% (13/77), hepatobiliary disorders at 14.3% (11/77), and gastrointestinal disorders at 13.0% (10/77). Regarding outcome, recovery, remission, non-recovery, and death were observed in 23.3% (21/90), 45.6% (41/90), 5.6% (5/90), and 6.7% (6/90) of the patients, respectively. The most common onset time was within 120 days at 40% (20/50) (Figs. 2a, 3a).

### Ethambutol

Eye disorders were the most common adverse events at 75.0% (60/80), followed by nervous system disorders (11.3%, 9/80). Regarding outcome, recovery, remission, non-recovery, sequelae, and death were observed in 8.3% (7/84), 13.1% (11/84), 35.7% (30/84), 7.1% (6/84), and 0.0% (0/90) of the patients, respectively. Particularly, in eye disorders, 44.6% (25/56) of patients did not recover, and 5.4% (3/56) were left with sequelae. The most common onset time was 181–300 days at 43.6% (21/48) (Figs. 2b, 3b).

### Rifampicin

Renal and urinary disorders were the most common adverse events at 26.5% (9/34), followed by investigations at 23.5% (8/34), and blood and lymphatic system disorders at 14.7% (5/34). Regarding outcome, recovery, remission, non-recovery, and death were observed in 47.5% (19/40), 22.5% (9/40), 2.5% (1/40), and 7.5% (3/40) of the patients, respectively. The most common onset time was within 120 days at 88.5% (23/26) (Figs. 2c, 3c).

### Reporting odds ratio

The RORs for CAM, EB, and RFP are shown in Table 1.

**Table 1:** Reporting odds ratio of adverse events of every drug

Drug	Adverse event	n	ROR	95% CI
CAM	Infections and infestations	13	4.13	2.3–7.44
	Blood and lymphatic system disorders	12	1.95	1.06–3.58
	Hepatobiliary disorders	11	2.61	1.39–4.91
	Gastrointestinal disorders	10	1.5	0.78–2.89
	Respiratory, thoracic, and mediastinal disorders	8	1.41	0.68–2.92
	Immune system disorders	6	2.38	1.04–5.44
	Metabolism and nutrition disorders	5	1.8	0.73–4.44
EB	Eye disorders	62	215.79	132.62–351.12
	Nervous system disorders	10	1.42	0.73–2.75
RFP	Renal and urinary disorders	9	7.03	3.35–14.77
	Investigations	8	6.99	3.22–15.18
	Blood and lymphatic system disorders	5	1.81	0.71–4.62

*CAM* clarithromycin, *EB* ethambutol, *RFP* rifampicin, *ROR* reporting odds ratio, *CI* confidence interval

For CAM, the RORs of infections and infestations, hepatobiliary system disorders, and immune system disorders were 4.13 (95% CI, 2.3–7.44), 2.61 (95% CI, 1.39–4.91), and 2.38 (95% CI, 1.04–5.44), respectively. For EB, the ROR of eye disorders was 215.79 (95% CI, 132.62–351.12). For RFP, the RORs of renal and urinary disorders and investigations were 7.03 (95% CI, 3.35–14.77) and 6.99 (95% CI, 3.22–15.18), respectively.

### Weibull distribution

Of the cases in which adverse events occurred in EB, the number of days of adverse events could be confirmed in 28 cases. We calculated the Weibull distribution of EB in the 28 patients with known onset of adverse events. The β value of EB was 2.07 (95% CI, 1.48–2.76), which was classified as a wear-out failure type (Table 2).

**Table 2:** Weibull distribution of ethambutol

Drugs	n	β	95% CI
EB	28	2.07	1.48–2.76

*EB* ethambutol, *CI* confidence interval

## Discussion

The Official ATS/ERS/ESCMID/IDSA Clinical Practice Guidelines recommend treatment with a 3-drug regimen, including a macrolide for macrolide-sensitive MAC-LD [17]. In this study, we firstly reported the problematic adverse events of treatments for MAC-LD by using a large database in Japan.

Of the 1200 adverse event reports, the total number of deaths, non-recovery, and sequelae was 19.4% (233/1200), which cannot be ignored. This result may be influenced by the bias that minor adverse events that are not clinically recognized are unlikely to be reported; therefore, severe adverse events may have been reported relatively more frequently. However, a systematic review showed that serious adverse events are also underreported [18]. Thus, we believe that serious adverse events, such as death, non-recovery, and sequelae, are common.

By calculating the ROR, we found that the most adverse events of CAM were infections and infestations, blood and lymphatic system disorders, hepatobiliary disorders, and immune system disorders. A study investigated the adverse events of switching from CAM to azithromycin (AZM) for MAC-LD when CAM was discontinued due to adverse events [19]. Patients who switched from CAM to AZM did not have more adverse events, and the most common adverse event that led to discontinuation was skin rash. Kadota et al. investigated the effect of CAM-based regimens for MAC-LD in Japan [20]. Adverse events occurred in 47.2% of CAM-based regimens, with 33.6% of adverse events related to CAM. The frequency of adverse events was not related to the total dose of CAM, doses per body weight, or age. The most common adverse events were skin rash, anorexia, hepatic dysfunction, diarrhea, and leukopenia. These results were not consistent with the results of this study because the regimens included drugs other than CAM, EB, and RFP. Kwon et al. compared the rate of treatment discontinuation due to adverse events of CAM and AZM for MAC-LD [21]. The most common adverse event that resulted in treatment discontinuation was gastrointestinal disorders. The frequency of adverse events varies among studies because of the difficulty of studying only CAM. However, infections, infestations, and immune system disorders may be related to adverse events, respiratory symptoms, or immunodeficiency caused by MAC-LD. The spontaneous reporting system does not fully explain the causal relationship and may be subject to reverse causality bias. In this study, the onset of adverse events within 120 days was 40%. This suggests that attention should be paid to the occurrence of adverse events early in treatment. This study is novel in that we show that adverse events of CAM tend to occur early in the course of treatment.

In this study, the most common adverse events occurred between 181 and 300 days after the start of treatment, indicating that attention should be paid to the adverse events of EB, especially 6 months after the start of treatment. Adverse events of EB develop depending on doses, and risk factors include high doses per body weight and daily administration [22–25]. In a case series of 70 patients with EB-induced eye toxicity, 36 patients developed eye toxicity between 2 and 6 months, and 26 patients developed eye toxicity more than 6 months after the start of treatment [26]. Therefore, the duration of treatment was also a risk. In a retrospective study of MAC-LD treatment in Japan, the median onset of eye disorders was as long as 278 days, which is a frequent cause of drug discontinuation (96.2%) [10]. In a retrospective review comparing patients who received standard treatment, including EB, and those who discontinued EB due to adverse events, the treatment failure rate tended to be higher in the group of patients who discontinued EB [14]. To avoid discontinuation of treatment, patients receiving EB should always be assessed for doses per body weight. In this study, 42.9% (36/84) of patients experienced non-recovery or sequelae, whereas 0.0% died. This may reflect the current situation in which patients are more aware of the adverse events of EB, are more regularly assessed for adverse events, and are more promptly treated when adverse events occur. In addition, using the Weibull distribution, we found that the onset time of eye disorder in EB was the wear-out failure type. This indicates that the incidence of adverse events increases over time. We should keep in mind that the treatment of MAC-LD requires long-term administration of EB and that the frequency of adverse events increases with the duration of treatment.

Using the ROR, we found that the most frequent adverse events when the suspect drug was RFP alone were renal and urinary disorders. In this study, we also found that most of the adverse events of RFP occurred within 120 days of administration. Few studies have examined the onset of adverse events of RFP alone, and we consider the results of this study to be important. Covic et al. investigated 60 cases of acute renal failure after RFP administration [27]. The amount that induced renal failure and the total dose were not related to the severity of renal failure, and dialysis was directly correlated with the length of anuria. The common histological finding was tubulointerstitial nephritis [27]. Muthukumar et al. reported 25 cases of acute renal failure due to RFP [28]. Symptoms in 24 patients developed between 1 to 24 h, which are consistent with this study’s result. Among 12 patients who underwent kidney biopsy, 7 patients had acute interstitial nephritis. In addition, 1 patient had crescentic glomerulonephritis, and 3 patients had mesangial proliferation. Another review of 48 RFP-associated renal failure cases also showed the most common histological finding was acute tubular necrosis [29]. Kim et al. reported a case who developed minimal change disease while taking RFP [30]. These findings revealed that RFP could cause various types of kidney injuries. Moussa et al. compared the blood concentration of RFP in patients with tuberculosis who experienced adverse events [31], and there was no difference in blood concentration. Since there may be little relationship between the dose and occurrence of adverse events, we should note decreased urine output and anuria regardless of the dose of RFP.

In this study, a comprehensive review of adverse event reports in the treatment of NTM allowed us to improve identifying adverse events that are likely to be caused by the drug. For example, we identified renal and urinary disorders associated with RFP, which will guide us to be attuned to these disorders during treatment. Since the study was negative on the usefulness of RFP for NTM [32], it can be used as an indicator for changing treatment when an adverse event occurs. We found that adverse events caused by CAM and RFP tended to occur within 120 days of treatment initiation, whereas those caused by EB tended to occur after 180 days. Monitoring the patient at specific times, depending on the drug, may reduce the need for discontinuation and, therefore, the occurrence of treatment failure. In addition, the use of the JADER database made it possible to determine the situation in Japan in general and to collect a large number of cases compared with previous studies that summarized adverse events.

This study has some limitations. First, spontaneous reporting systems do not contain data on the total number of patients that use a drug. Therefore, signal detection by disproportionality analysis frequently focuses on differences in the ratio of adverse event reports [33]. These results may be affected by unverified and incomplete reports, underreporting, and indication and reverse causality biases. Small sample sizes may increase these biases. Second, a previous study showed that the quality of reports differed depending on the facilities or occupations in the JADER database [34], and the quality of the reports was not evenly distributed. Third, although ROR is suggested to be less biased than the proportional reporting ratio, which is a ratio of reporting proportions, there are still various biases [35]. We need to accumulate more cases to understand the actual situation of adverse events. Other data mining methods include the Gamma Poisson Shrinker program and Bayesian approaches such as the Bayesian Confidence Propagation Neural Network, which were not used in this study [36]. Fourth, some reports did not describe specific outcomes, and there was a discrepancy between the number of adverse events and outcomes. In addition, multiple events may have been reported more than once at the same lesions. Fifth, there are several interaction signals [37, 38], but since MAC-LD treatment is in principle a three-drug combination, signals specific to interaction cannot be detected in this study. Sixth, the number of days of adverse events of EB was reported in 28 of 62 patients. Consequently, the analysis of the Weibull model may be inaccurate because of the large number of missing values. Seventh, we identified the most frequently reported sex and age for each drug. However, since we did not analyze non-cases, we could not perform a statistical analysis to determine if sex and age were risk factors. We need to accumulate more cases to understand the actual situation of adverse events.

## Conclusions

By calculating the ROR, we clarified the actual adverse events of CAM, RB, and RFP. We should monitor infections and infestations as well as hematologic, lymphatic, hepatobiliary, and immune system disorders with CAM. Eye disorders are a concern with EB treatment, while RFP requires attention for renal and urinary disorders. We also clarified the time to adverse events for CAM, EB, and RFP. For MAC-LD, adverse events of EB occur after 180 days, whereas the adverse events of CAM and RFP occur earlier during the treatment course. Depending on the drug, monitoring the patient at specific times may reduce the need for discontinuation and, therefore, the occurrence of treatment failure.

## Data statement

This study was conducted using the Japanese Adverse Drug Event Report (JADER) database provided by the Pharmaceuticals and Medical Devices Agency (PMDA). The information, results, and interpretation of this study do not constitute an expression of any opinion of the PMDA.

## Supplementary Information

**Additional file 1: Fig. S1**. Number of adverse events in *Mycobacterium avium* complex lung disease in the Japanese Adverse Drug Event Report database. MAC-LD *Mycobacterium avium* complex lung disease

**Additional file 2: Fig. S2.** Patient’s characteristics in *Mycobacterium avium* complex lung disease (MAC-LD) in the Japanese Adverse Drug Event Report database. Horizontal axis shows the number of MAC-LD cases. Each bar chart is divided by the number of cases according to age.

**Additional file 3: Fig. S3**. Outcome of less than 20 adverse events in all three drugs: clarithromycin, ethambutol, and rifampicin. Vertical axis shows the types of adverse events. Horizontal axis shows the proportion of cases by adverse event. Each bar chart is divided by the number of cases according to outcome.

**Additional file 4: Fig. S4**. Age and sex of cases who experienced adverse events in each drug. Vertical axis shows sex. Horizontal axis shows the number of patients who experienced adverse events by sex. Each bar chart is divided by the number of patients according to age. (a) Clarithromycin. (b) Ethambutol. (c) Rifampicin.

**Additional file 5: Fig. S5**. Weibull distribution of adverse events in Mycobacterium avium complex lung disease. Vertical axis shows the cumulative probability. Horizontal axis shows the day of the onset of adverse events. (a) Clarithromycin. (b) Ethambutol. (c) Rifampicin.

## Data Availability

The datasets generated and/or analyzed during the current study are available in the Japanese Adverse Drug Event Report (JADER) repository, http://www.info.pmda.go.jp/fukusayoudb/CsvDownload.jsp.

## Abbreviations

AZM: Azithromycin
CAM: Clarithromycin
EB: Ethambutol
JADER: Japanese Adverse Drug Event Report
MAC-LD: *Mycobacterium avium* Complex lung disease
NTM-LD: Nontuberculous mycobacterial lung disease
PMDA: Pharmaceuticals and Medical Devices Agency
RFP: Rifampicin
ROR: Reporting odds ratio
