# Supplementary figures and images for: Analysis of adverse drug events in pulmonary Mycobacterium avium complex disease using spontaneous reporting system
Source: BMC Infect Dis. 2022 Jun 29;22:580. doi: 10.1186/s12879-022-07568-z (PMC9241207; doi:10.1186/s12879-022-07568-z)

MAC-LD  
806 patients (2780 cases)

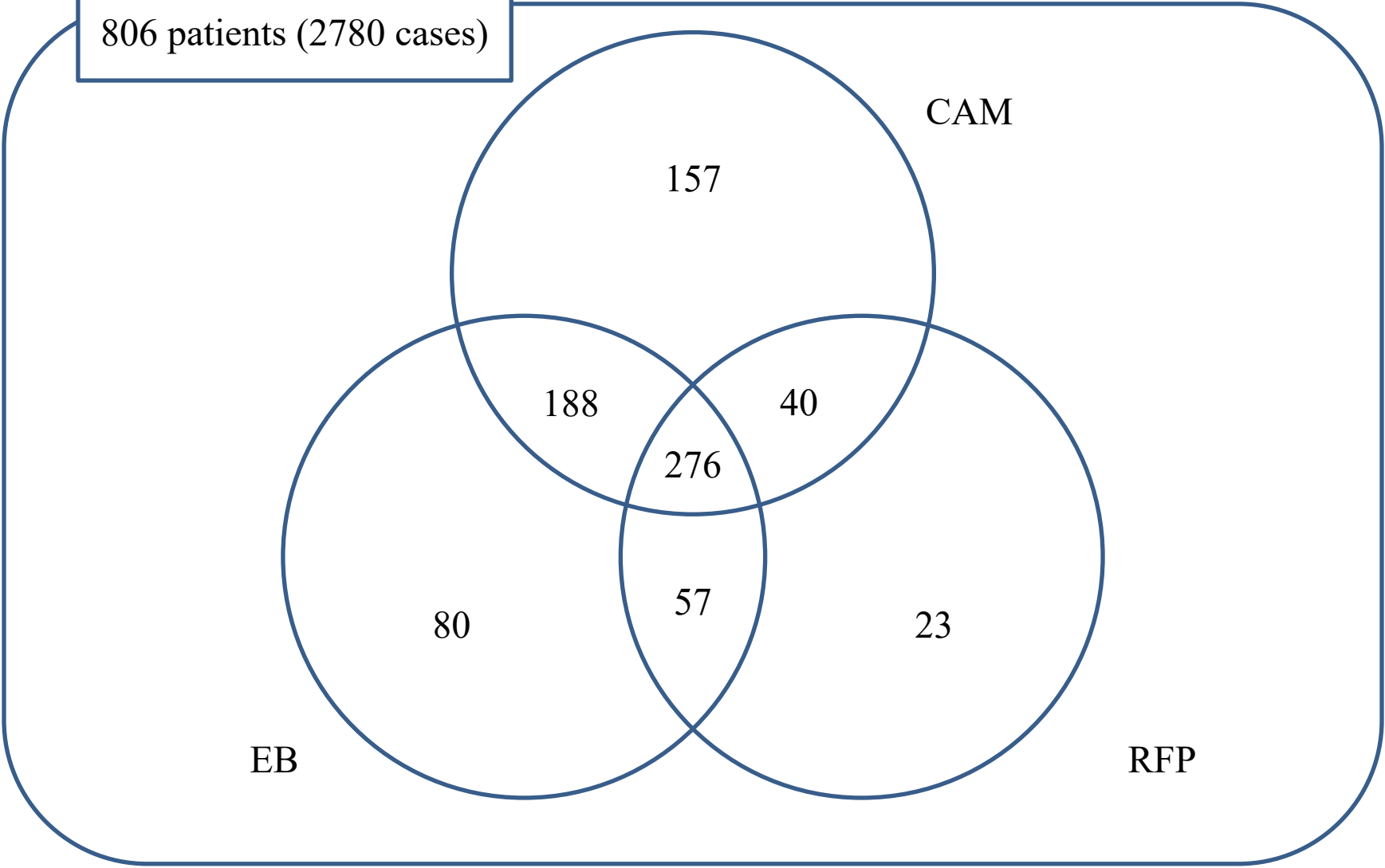

Supplement: Supplementary file 1 — Additional file 1: Fig. S1. Number of adverse events in Mycobacterium avium complex lung disease in the Japanese Adverse Drug Event Report database. MAC-LD Mycobacterium avium complex lung disease [file 12879_2022_7568_MOESM1_ESM.pdf]

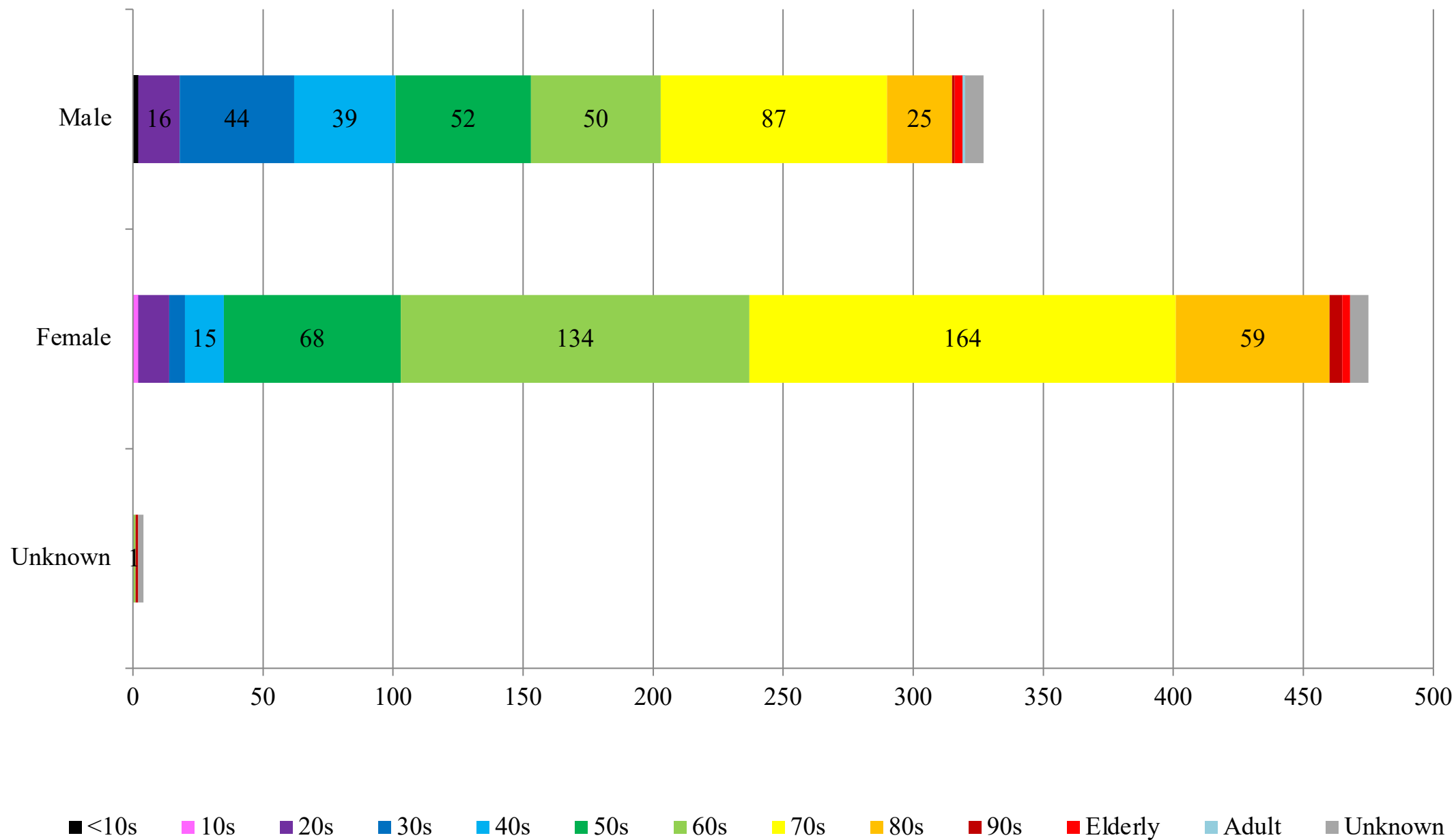

Supplement: Supplementary file 2 — Additional file 2: Fig. S2. Patient’s characteristics in Mycobacterium avium complex lung disease (MAC-LD) in the Japanese Adverse Drug Event Report database. Horizontal axis shows the number of MAC-LD cases. Each bar chart is divided by the number of cases according to age. [file 12879_2022_7568_MOESM2_ESM.pdf]

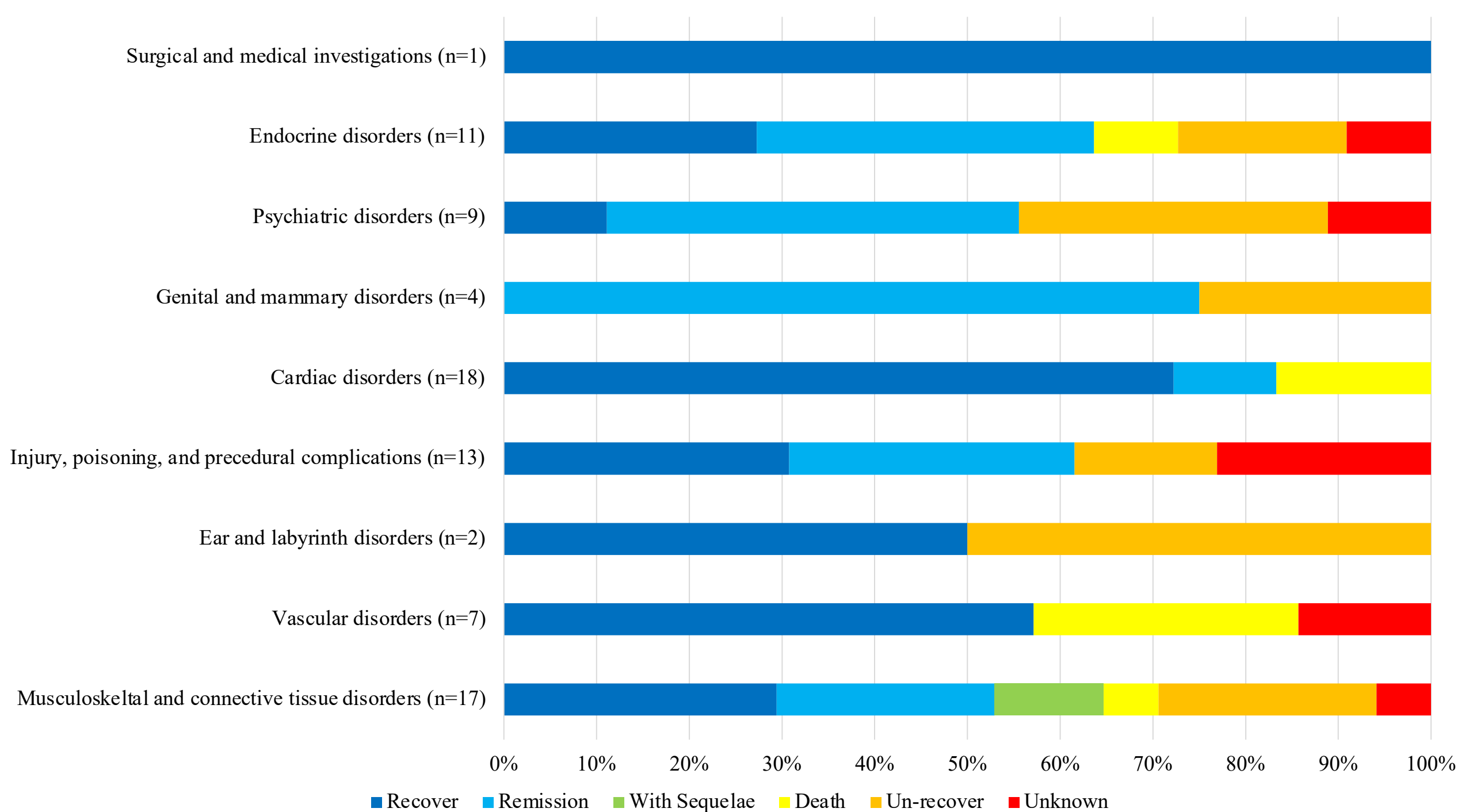

Supplement: Supplementary file 3 — Additional file 3: Fig. S3. Outcome of less than 20 adverse events in all three drugs: clarithromycin, ethambutol, and rifampicin. Vertical axis shows the types of adverse events. Horizontal axis shows the proportion of cases by adverse event. Each bar chart is divided by the number of cases according to outcome. [file 12879_2022_7568_MOESM3_ESM.pdf]

a

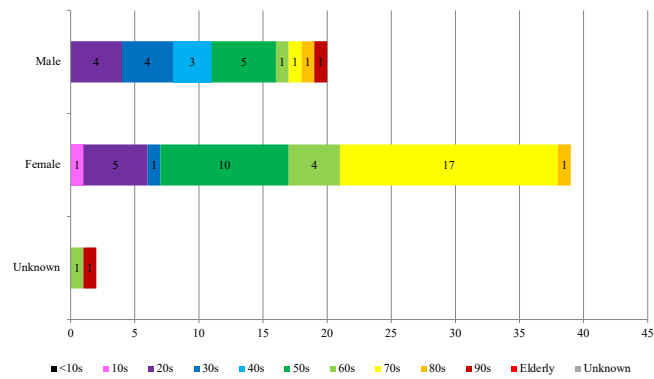

b

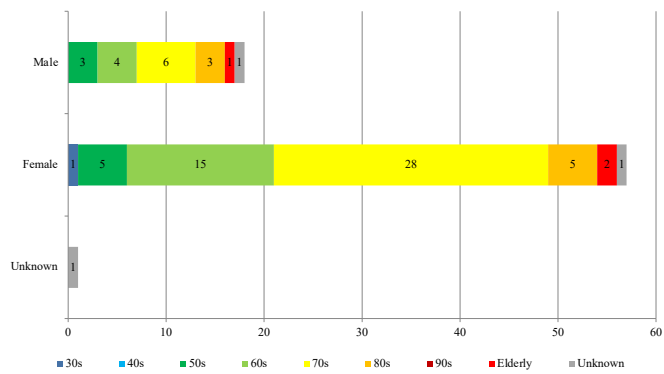

c

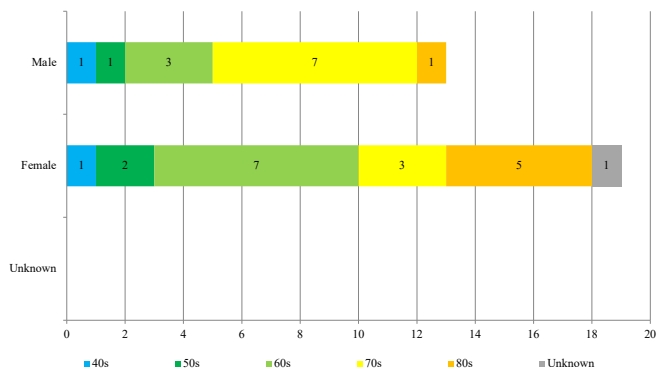

Supplement: Supplementary file 4 — Additional file 4: Fig. S4. Age and sex of cases who experienced adverse events in each drug. Vertical axis shows sex. Horizontal axis shows the number of patients who experienced adverse events by sex. Each bar chart is divided by the number of patients according to age. (a) Clarithromycin. (b) Ethambutol. (c) Rifampicin. [file 12879_2022_7568_MOESM4_ESM.pdf]

a

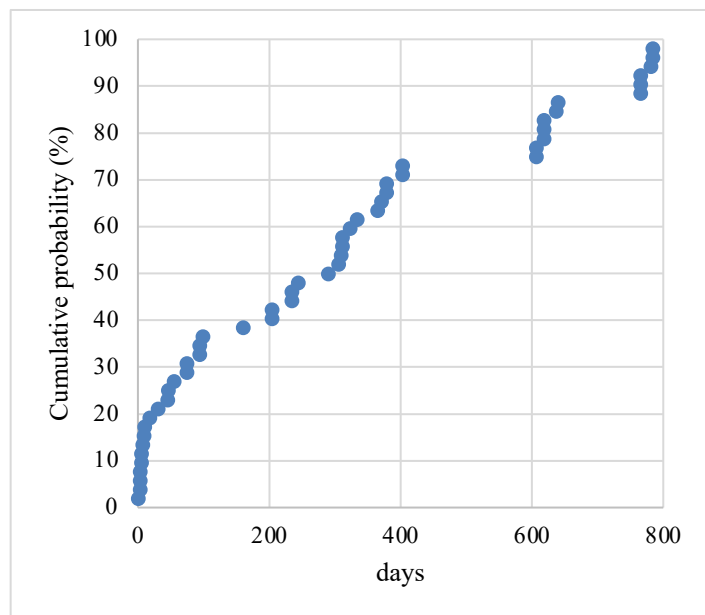

b

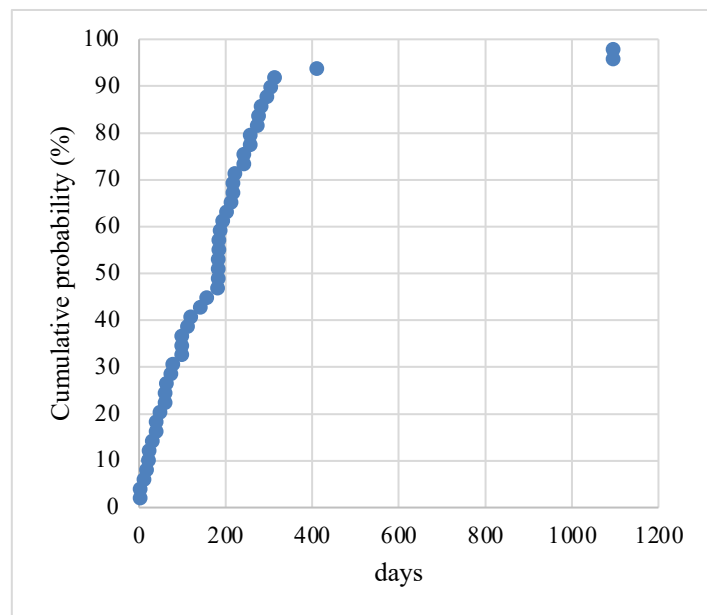

c

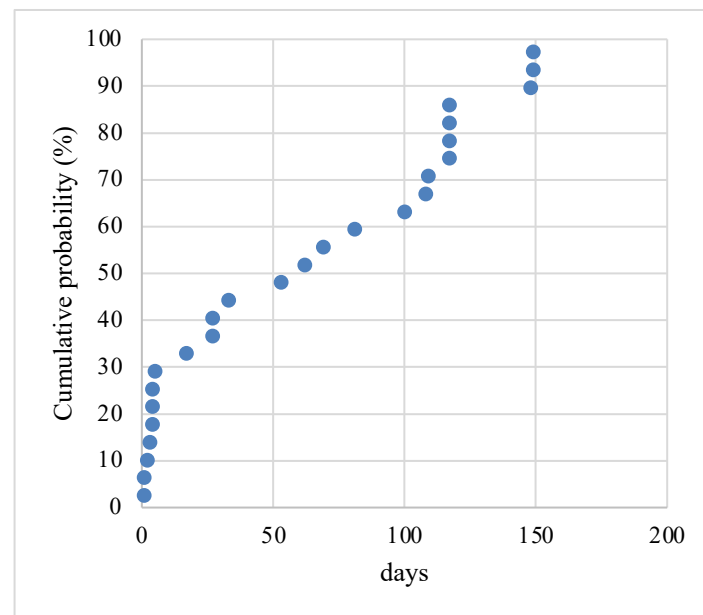

Supplement: Supplementary file 5 — Additional file 5: Fig. S5. Weibull distribution of adverse events in Mycobacterium avium complex lung disease. Vertical axis shows the cumulative probability. Horizontal axis shows the day of the onset of adverse events. (a) Clarithromycin. (b) Ethambutol. (c) Rifampicin. [file 12879_2022_7568_MOESM5_ESM.pdf]
